# Exploring the Role of Non-Structural Carbohydrates (NSCs) Under Abiotic Stresses on Woody Plants: A Comprehensive Review

**DOI:** 10.3390/plants14030328

**Published:** 2025-01-22

**Authors:** Ayesha Fazal Nawaz, Sara Gargiulo, Alessandro Pichierri, Valentino Casolo

**Affiliations:** 1 Department of Life Sciences, University of Trieste, via L. Giorgieri 10, 34127 Trieste, Italy; ayeshafazal.nawaz@phd.units.it (A.F.N.); alessandro.pichierri@phd.units.it (A.P.); 2 Dipartimento di Scienze Agroalimentari, Ambientali ed Animali, Università di Udine, via delle Scienze 206, 33100 Udine, Italy; sara.gargiulo@uniud.it

**Keywords:** non-structural carbohydrates (NSCs), woody plants, abiotic stress, drought, salinity, heat, cold, waterlogging, plant growth

## Abstract

Global climate change has increased the severity and frequency of abiotic stresses, posing significant challenges to the survival and growth of woody plants. Non-structural carbohydrates (NSCs), including starch and sugars, play a vital role in enabling plants to withstand these stresses, helping to stabilize cellular functions by buffering plant energy demands and facilitating recovery on the alleviation of stress. Despite the recognized multiple functions of NSCs, the contrasting effects of multiple abiotic stresses on NSCs dynamics in woody plants remain poorly understood. This review aims to explore the current knowledge of the contrasting effects of abiotic stress conditions including drought, salinity, heat, water logging, and cold on NSCs dynamics. The roles of NSCs in regulating stress-resilience responses in woody plants are also discussed, along with the challenges in NSC measurement, and options for future research directions are explored. This review is based on comprehensive literature research across different search engines like Scopus, Web of Science, and Google Scholar (2000–2024) using targeted keywords. This study compiles the current research on NSCs functions and provides insights into the adaptive strategies of woody plants in response to changing climate conditions, providing groundwork for future research to improve stress tolerance in woody plants.

## 1. Introduction

Long-lived woody plants face various abiotic stresses that can be repeated during their life, adversely affecting the overall plant health [1]. Among them, drought, salinity, low temperatures, waterlogging, and heat represent a serious threat to both the sustainable agriculture sector and the natural ecosystem [2,3]. Extensive research has been conducted on the individual effects of abiotic stresses on Non-structural carbohydrates (NSCs) dynamics as Adams et al. [4] explained altered NSCs mobilization and partitioning under drought stress. Similarly, other studies explored the roles of NSCs in intensifying the osmotic stress responses under salinity [5] and the impacts on root carbohydrate storage under waterlogging stress [6]. Different studies also explained the cryoprotective and homeostatic roles of NSCs under extreme temperatures [7,8]. However, the similarities and differences in NSCs responses under these stress conditions remain unclear.

Climate constraints are expected to increase in the coming decades with significant effects on plants’ growth and their capacity to adapt to the evolving environmental conditions [9,10]. In this light, increases in drought and extreme heat strongly affect the global hydrological cycle [11] and the precipitation pattern across the year. Indeed, precipitation is anticipated to decrease significantly, leading to a high risk of drought events during the summer period [12]. The effective drying in some regions leads forests to face the risk of the mortality of dominant tree species [13].

Indeed, under drought stress, stomatal closure minimizes the transport of water through the xylem and the uptake of carbon dioxide, leading to a decline in photosynthetic activity and a reduction in carbon assimilation. This ultimately hinders the translocation of sugars to the phloem. The disruption of this system affects the storage of NSCs and, ultimately, interacts with hydraulic functioning, leading to plant mortality [4,14].

The ability to resist abiotic stress is essential for the long-term survival of plants and is in part governed by the process of carbon (C) storage and allocation [15]. NSCs are photosynthetic products that are used as a building block for plant growth and other anabolic processes [16]. Primarily, NSCs are stored in the stem and roots in the form of starch and sugars before heading to the main organs when requested [8]. The assimilation, transport, and distribution of carbohydrates from the source organs (mainly leaves) to the sink organs (seeds, fruits, roots, and stems) is essential to satisfy plant needs and activate the response to abiotic stresses [17]. Although the functions of these different carbohydrates are far from being fully understood, it is known that they may serve multiple roles as cryoprotectants, signaling molecules, osmotic regulators, and scavengers of reactive oxygen species (ROS) [18] to protect plants against environmental stress [19]. NSCs are accumulated during periods of surplus supply from photosynthesis and constitute an important buffer during environmental stress when carbon demand outstrips supply [20]. In particular, soluble NSCs like fructose, glucose, sucrose, and sugar-alcohols (e.g., sorbitol, mannitol) are essential to act as osmoprotectants and maintain cell turgor under osmotic stress, e.g., salt and drought stress [21,22].

Thus, understanding the regulation of C allocation is essential for predicting the plant responses to environmental changes [23] in both herbaceous perennials [24] and woody crops [25].

(i) Herbaceous crops. In herbaceous plants, the abiotic stress factors have adverse impacts as they accelerate flowering and reduce the grain-filling period in the Poaceae family, resulting in a smaller grain set, weight, and size [26]. Studies examining the composition and content of reserve compounds in herbaceous species revealed the presence of notable quantities of fructans along with other soluble carbohydrates such as glucose, fructose, oligosaccharides, and sucrose [27]. Following drought stress and, thus, photosynthesis limitation, the recovery of herbaceous plants requires carbon, and it must come from the mobilization of NSCs reserves [28] often allocated in the underground reserve organs [29]. A recent study proposed that the fructans play an important role in drought resilience across grass species [30]. The water-soluble nature of fructans contribute in the osmotic adjustment of cells by altering the degree of polymerization of molecules and serving a protective function for cell membranes during dehydration [31]. However, understanding the role of fructans in abiotic stresses is a very complex process that cannot be fully elucidated by the action of a single molecule or mechanism. The role of fructans in stress resilience is influenced by several factors such as differences in molecular size, chemical structure, and cell localization [28]. In general, the responses and roles of NSCs remain understudied in the context of herbaceous perennials, especially during the recovery phase [24].
(ii) Woody crops. In contrast to herbaceous species, woody plants rely less on the mobilization of short-term NSCs reserves for immediate recovery from stress conditions and more on the long-term NSCs reserves to withstand prolonged stress conditions and tissue recovery [29,32]. The storage parenchyma present in the roots and stems of woody species possess relatively high constitutive NSsC pools that enable these long-lived plants to overcome periods of disturbance and stress [33]. The development of trees and NSCs reserves is strongly correlated as NSCs are invested in growth or accumulated in storage organs to enhance tree resilience, depending on climatic inputs [34]. The response of NSCs varies across species and among different organs, even under the same environmental conditions. Additionally, the translocation of NSCs is necessary to overcome hydraulic dysfunction under water limitation, but, in such critical conditions, carbon starvation can occur due to a prolonged low photosynthetic rate and prevent NSCs supply [35]. Hydraulic dysfunction arises when plants do not receive sufficient water, and plants utilize NSCs, particularly sugars, to maintain the hydraulic system and structural integrity [33]. Many studies indicate that under stress conditions the stored starch degrades into sugars that act creating an osmotic gradient to drive the water flow toward the embolized xylem for refilling [36].

The frequent occurrence of stresses can lead to the depletion of NSCs stored by plants for subsequent utilization [37], preventing plant functional processes, including osmoprotection, osmoregulation, and cryoprotection, during abiotic stress [38]. However, an updated comprehensive report of the main dynamics describing physiological responses to different abiotic stress is needed. This review aims to assess (i) the implications of NSCs dynamics under changing environmental conditions; (ii) the similar and divergent effects of abiotic stresses (i.e., drought, salt, waterlogging, heat, and cold) on NSCs dynamics in woody plants; and (iii) how NSCs regulate stress-inducing signaling pathways and influence plant physiological functions; (iv) and to identify the NSCs research challenges and a framework for future research.

## 2. Abiotic Stresses and Effects on Crops: Drought, Salt, Waterlogging, Heat, Chilling (Low Temperatures)

**Figure 1:**
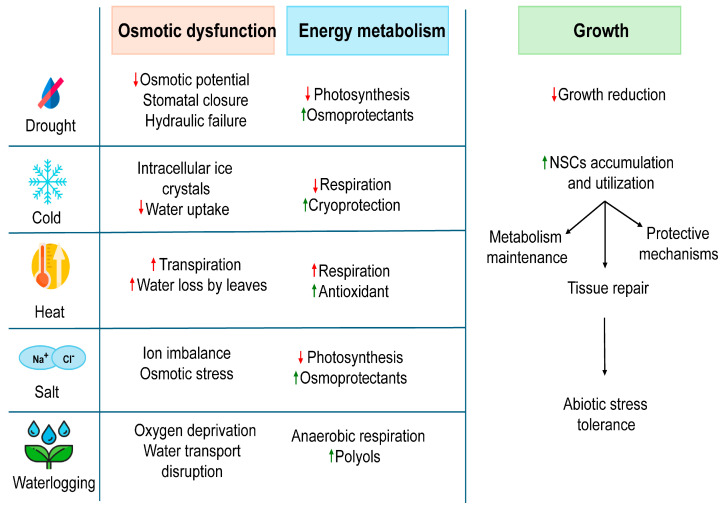
Effects of abiotic stress on plant functions and implications of NSCs for stress tolerance. The changes in different physiological parameters in response to specific stress are indicated by arrows as upward red arrows representing increase and downward red arrows indicating decrease. Green arrows highlight the positive responses for the accumulation of stress-protective compounds.

Climate change has intensified abiotic factors challenging in perennial plants’ and influencing their ability to endure such stress for long-term survival, which is partly governed by the carbon storage and allocation process [16]. Here, we explore the impact of abiotic stress on woody plants and examine the role of NSCs in mitigating these stresses (Figure 1). 

### 2.1. Drought

Over the past decades, prolonged drought events have led to tree mortality in woodland communities [39]. The drought-induced mortality mechanisms involves hydraulic failure due to a disturbance in water regulation and also associated with the impairment of C-dependent hydraulic and metabolic processes [40,41]. Xylem is the main pathway for long-distance water transport in trees and works in a metastable state, which can be interrupted by the formation of air bubbles leading to an embolism [42]. Under water stress conditions, air bubbles from the embolized conduits can spread to adjacent conduits through pits and damage the continuous water column, which ultimately disrupts water transport, leading to hydraulic failure in the xylem and eventually to tree mortality [43,44]. Plants can be divided into two categories according to the hydraulic strategy adopted to cope with water shortage: isohydric and anisohydric. Isohydric plants immediately close the stomata to save water losses due to evapotranspiration but must deal with the lack of photoassimilates. On the contrary, anisohydric plants are considered drought-tolerant because they maintain the stomata opened to preserve photosynthesis activity. However, the low water potential induced by severe drought conditions leads to a critical water status, increasing the risk of hydraulic failure [45].

Drought-induced tree mortality may be exacerbated by carbon starvation caused by the depletion of stored carbohydrates [41]. Embolism-avoidance mechanisms cause stomatal closure; lead to a decrease in photosynthetic activity; and, when the carbon consumption of plants exceeds the carbon assimilation [46], cause carbon starvation [47]. This carbon starvation might hinder the mechanism associated with the refilling of embolized conduits, thereby worsening the water transport capacity [48]. Insufficient NSCs levels in the secondary phloem can lead to the loss of phloem turgor and the subsequent leakage of water into the xylem [49]. Additionally, carbon starvation might hinder the production of metabolites essential for plant defense and make the plants more susceptible to biotic agents that can further block the xylem vessels [40,47].

**Figure 2:**
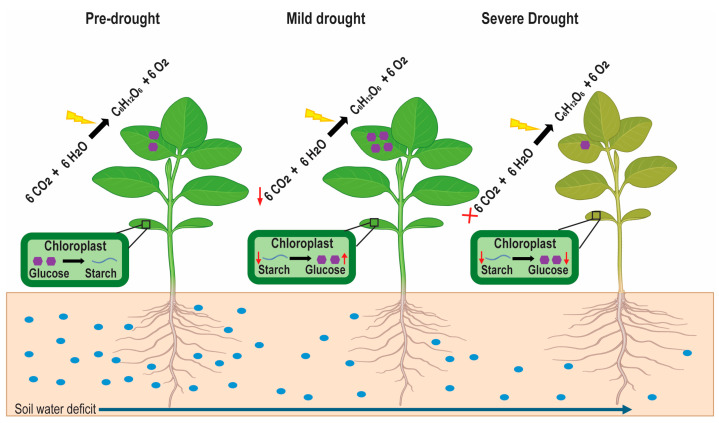
Variation in photosynthesis, starch, and glucose dynamics under different drought conditions. Pre-drought conditions show a normal photosynthesis rate with regular glucose concentration and starch accumulation. In mild drought conditions, the photosynthesis rate declines (downward red arrow), increasing starch degradation to maintain high glucose levels (upward red arrow) and lower the starch reserves (downward red arrow). Severe drought ceases photosynthesis (red cross), resulting in minimal glucose and starch levels (respective red arrows).

Under drought stress, the quantity and distribution of NSCs are altered. Initially, during a short period of drought stress, plant photosynthesis is inhibited, and a reduction in the photosynthetic rate occurs after a decline in the growth rate, resulting in the accumulation of NSCs within plants [50,51]. However, prolonged drought stress disrupts the balance between carbon uptake and carbon consumption in plants [52]. Consequently, the level of glucose and starch decreases (Figure 2), and the plant requirements for growth, respiration, and defense mechanisms cannot be fulfilled [46,53].

NSCs play a crucial role in enhancing plant resistance to drought stress. Indeed, to deal with drought stress, plants accumulate a certain level of NSCs as supplementary energy reserved for water stress resistance [54]. This storage can help to balance the reduced photosynthetic supply and metabolic demand, allowing the trees to endure drought conditions [53]. Generally, sugar accumulation under drought stress plays a protective role by inhibiting the oxidative damage to the cell membranes while maintaining membrane hydration and turgor [55]. For instance, the multiple functions of glucose such as energy supply, cellular stabilization, signaling molecule, and inducing stomatal closure to conserve water under adverse conditions help the plants to achieve drought tolerance [56]. In some plants, fructose, glucose, and sucrose also serve to support root growth, which play important roles in water uptake in the deepest soil layers [57]. Starch is also essential for drought tolerance as it can be hydrolyzed and mobilized to provide a continued supply of sugars to maintain physiological functions, adjust the osmotic balance, and mitigate C starvation [53]. Starch is also important for post-drought recovery, as its remobilization allows the quick restoration of plant growth by providing sources to repair tissue damage, helping the plant’s fast recovery [58]. Additionally, there are certain raffinose family oligosaccharides (RFOs) like stachyose, verbascose, and raffinose that help the plant to maintain cell hydration and provide osmoprotection and antioxidant defense under drought stress [59]. Sugars, along with sugar alcohols, inorganic ions, and amino acids accumulated to regulate the osmotic potential in living cells including secondary phloem and wood parenchyma to maintain the cell turgidity under low water conditions [44,46]. Plants with higher NSCs levels maintained greater stem water potentials under drought stress and had better survival rates [60].

### 2.2. Salt

Salinity is a widespread environmental challenge for plants, characterized by the high concentration of salts in the soil, which adversely affects plant biomass production and agricultural economies [61]. Globally, salinity is reported to negatively impact 11% of the world’s total irrigated land [62]. A plant’s mineral nutrition is affected by salt stress as it inhibits the uptake of essential nutrients such as Ca^+2^ and K^+^ from the soil, and it also causes oxidative stress through the generation of ROS [63]. In general, different plants have varying levels of susceptibility to salinity, which causes ionic and osmotic stress [64].

With the increase in the salt-affected land area, the ability of plants to tolerate salt stress is a highly valuable agricultural trait [65]. Plants have developed tolerance mechanisms to cope with salt stress, including physiological mechanisms to help the plants in the maintenance of good water status and transpiration efficiency [5]. For example, the synthesis and accumulation of compatible solutes sustains osmotic adjustment and plays a key role in protecting subcellular structures from oxidative damage. Moreover, plants living in saline environments have adopt adaptive strategies like ion compartmentation, leaf turnover, and salt glands [61]. Halophyte plants inhabiting salt-affected soils are characterized by high resistance to salt stress because of adaptive salt-tolerant morphophysiological mechanisms [66]. This salt tolerance is associated with the accumulation of polyols and NSCs [6]. Primarily, sugars and sugar alcohols increase to serve as osmolytes [67], contributing to the maintenance of cell homeostasis and enhancing antioxidant protection [68]. Indeed, the accumulation of sugars produces hydration shell structures around macromolecules and helps to prevent the peroxidation of cells [69]. Moreover, different sugars and their derivatives including sucrose, maltose, mannobiose, glucose, fructose, melibiose, and trehalose also showed upregulation to overcome the salt stress [70]. For example, the content of neoagarotetraose and agarobiose increases in the leaves and roots to counteract the toxic effects of salt stress [69].

### 2.3. Waterlogging

Waterlogging caused by surplus water in the soil leads to a decrease in the oxygen level in the soil and hinders the ability of plants to absorb nutrients, affecting the plants’ growth and yield [71]. Except for a few species such as mangroves, which have adapted to waterlogged environments [72], most trees in their native habitat are not well adapted to submerged life. Waterlogging hinders the roots’ growth and function because of a shortage in oxygen levels that induces the accumulation of organic acids in the soil, inhibiting the production of respiration substrates [73] and, therefore, limiting the roots’ respiration [74,75]. Additionally, it limits light access to plants, hindering the photosynthetic ability of plants and resulting in the retardation of plant growth due to the energy and carbon crises [76]. Consequently, a significant reduction in NSCs concentrations is recorded in the leaves and roots of waterlogged trees [77], suggesting a severe impairment in the transport of sugars from the phloem to the roots under waterlogged conditions [78].

To survive under waterlogging conditions, the roots need to have an adequate level of NSCs [79]. However, an accumulation of sugars and starch in stems and roots is observed [80], likely due to the active allocation of carbon to reserve formation and the inhibition of the use of NSCs for the growth and development of plants [81]. Starch is an essential nutrient reservoir acting as a respiratory substrate, supporting plant tolerance of and recovery from waterlogging [77]. There is experimental evidence that waterlogging-resistant trees have higher levels of carbohydrate concentrations than waterlogging-sensitive species [78]. Indeed, the demand for soluble carbohydrates in the roots of waterlogging-tolerant species is partially fulfilled by the breakdown of stored starch reserves [79] as it contains highly active amylases, which can help to prevent soluble-carbohydrate shortage during submersion [82].

Under waterlogging conditions, to reduce the production of harmful substances in the soil, like ethanol, plants rely on their secondary metabolic pathways such as enhancing the metabolism of malic acid, tartaric acid, and oxalic acid [83,84]. NSCs play essential roles in the secondary metabolism of these organic acids and help to mitigate the negative impacts of flooding on plants [84].

### 2.4. Heat

Global warming is anticipated to be more severe over the next two decades with 1.5 °C of average annual temperature rise if greenhouse gas emission is sustained at the current level [85]. This warm climate is expected to lead to more adverse heat stress, which will negatively impact most of the cellular processes involved in plant growth and productivity [86]. Heat stress damages the photosynthetic system, in particular, photosystem II (PSII), altering the CO_2_-fixation system, the electron transport chain, photophosphorylation, and the oxygen-evolving complex [87]. Moreover, high temperatures induce stomatal closure and, consequently, decrease gas exchange, interfering with the plant’s water relations [88]. These constraints influence carbon portioning among plant organs. On one side, respiration rates increase and carbon consumption may exceed the carbon uptake, which ultimately leads to the potential depletion of NSCs reserves [86]. On the other side, stomatal closure can initially promote NSCs storage in the roots [88]. The level of heat damage is influenced by different factors like plant inherent heat sensitivity, developmental stage and the intensity, and duration of heat stress [89]. There are certain high-yield crops that leads to growth losses when heat stress occurs during the gametogenesis and flowering stage in comparison to stress exposure after the flowering stage [90].

Furthermore, NSCs are essential in alleviating long-term damage to the plants [91], the synthesis of specific carbon compounds such as sugar alcohols also helps to prevent protein denaturation at high temperatures and to maintain cellular functions [88]. In agriculture, heat-tolerant cultivars show a high level of NSCs in their stems at the heading stage and utilize an escape mechanism by boosting the starch metabolism to compensate for the shortening of the grain-filling stage and enhancing the sucrose uptake by the upregulation of sucrose transporters [92]. Under heat stress, certain heat-tolerant rice cultivars maintain a higher level of NSCs in their stems to facilitate the swift transfer of NSCs from the stems to the grain to contribute to a greater yield [93].

### 2.5. Cold (Low Temperatures)

Cold stress is one of the most critical abiotic stress factors that restrict crop growth and development. It also has major limitations on the geographical distribution of plants [94]. Cold stress can be categorized into chilling stress (0–10 °C) and freezing stress (below 0 °C) [95]. Chilling stress has adverse effects on the relative metabolic pathways of plants by disrupting membrane protein activity and stability [96]. It also leads to the accumulation of reactive oxygen species, leading to the deactivation of proteins, the disruption of nucleic acid structure, and membrane lipid peroxidation, which impair the cellular functions and overall plant growth [97]. Freezing stress can cause more severe damage as it leads to ice core formation inside and outside of the cells and causes cellular dehydration, leading to the destruction of cell structures [98]. In adverse cases, these ice crystals can puncture plant cells, leading to cytosol leakage and eventually plant death [7]. In regions with temperature fluctuations, plants experience repeated freezing and thawing events. These freeze–thaw cycles in their xylem tissues can cause an embolism [99], leading to hydraulic dysfunction [100]. This occurs because, as the sap freezes, bubbles are formed by the dissolved gases in the sap, and, during thawing, these bubbles may enlarge to embolize the conduits, leading to the disruption of water transport [101]. Long-term exposure to cold stress causes membrane lipid phase changes and enhances membrane permeability, which leads to increases in the leakage of cellular components and crucial electrolytes like carbohydrates, unsaturated fatty acids, amino acids, and metabolic compounds and causes osmotic stress. A disruption in the ion balance between the inside and outside of the cell can result in cell damage or death [94].

The most common strategy used by plants to cope with cold stress is acclimation by accumulating cryoprotective osmolytes including sugars, which enable the plants to survive under freezing conditions. Cold-stress-tolerant plants typically show a higher level of sugars like glucose, amylose, glucose 6 phosphate, maltose, and starch in their underground tissues [7,102]. Woody plants store NSCs as starch in the parenchyma cells of the roots and wood during the growing season to support the winter survival of plants and, in spring, for the development of new tissues and organs [25]. Under cold stress, stored starch is converted into soluble sugars to decrease the osmotic potential of cells, which helps to lower the freezing point of plant living cells and to prevent ice damage [103]. In response to low temperatures, the increase in starch degradation is influenced by enzyme activity and metabolic gene expression to regulate the onset tolerance mechanism [104]. Plants use NSCs for frost resistance, respiration maintenance, embolism refilling, membrane stabilization, and scavenging of ROS [25].

The management of NSCs within a tree is highly responsive to soil and atmospheric temperature variations [103]. During the period of early winter or fall, when the soil temperature is higher than the atmospheric temperature, the NSCs tend to accumulate in the roots. Conversely, during the period of early summer or spring, when the atmospheric temperatures are higher than the soil temperatures, the NSCs are directed to store in above-ground biomass. This pattern suggests the influence of temperature on NSCs distribution across the whole plant (roots to shoots) [25].

Also, snow cover determines chilling conditions, making it necessary for plants to manage a sufficient store of carbohydrates to perform cold hardening [105]. Recently, it has been demonstrated that snow cover can shift the starch/sugar ratio [106] and trigger the accumulation of starch and pinitol in plant organs [107]. However, a mechanistic interpretation of the phenomenon was given only in common juniper [108], which demonstrated a trade-off between snow cover, plant growth, and NSCs accumulation in bark.

## 3. NSCs Research Challenges and Future Directions

The research on NSCs under abiotic stress has predominantly focused on widely distributed genera such as *Populus* [109], *Quercus* [110], *Picea* [111], *Pinus* [112] and other forest trees typical of temperate forests. Meanwhile, the understanding of NSCs functional roles in woody crops and in other species that could be crucial for sustaining ecological biochemical cycles in certain biomes remains underexplored. Therefore, it is crucial to design comparative studies for a wide range of plant species and stress types to elucidate the clear patterns for the adaptive roles of NSCs as proposed by Uscola et al., 2015 [113]. The mobilization of NSCs during stress and recovery is linked to the plant phenology and to other factors like climatic conditions, plant age, developmental stage, and growth patterns [113,114]. Therefore, a key challenge in understanding the dynamics of NSCs across different species also involves identifying the specific plant tissues to target, optimal timings for tissue collection and in determining the variability in the dynamic responses of NSCs to different stress conditions, which could have significant impacts on the outcome of studies.

The allocation of NSCs across various plant tissues such as roots, leaves, branches, and stems plays a critical role in stress conditions, but it remains a complex topic to be unraveled by ecologists [115,116]. A review on NSCs highlighted that starch is typically reserved in the roots and, under stress conditions, could be transported to the leaves to be converted to sugars to maintain the turgor pressure [29]. Additionally, tissue-specific studies of NSCs in pine forests revealed that there are no effects of drought on the NSCs level in the shoots [117]. But, in another study, it was found that, in *Betula platyphylla* and *B. sylvestris* var. *mongolica*, the allocation of starch to the shoots is an immediate response under stress conditions [115]. In addition, in tall trees, the mobilization of NSCs from source to sink organs may cause impairments due to long pathway resistance and phloem viscosity, causing uneven NSCs distribution across plant organs [118]. This dynamic nature of NSCs often complicates the identification of the general pattern and poses major obstacles to conducting reliable meta-analyses. Many studies have explained the interconversions of the NSCs pools (i.e., sugars and starch), but there is no generalized prediction for specific types of NSCs to use as metabolic substrates and osmotic regulators under specific stress conditions. Chemical species of NSCs used for energetic demands, under specific conditions, can perform other roles, such as fructose and sucrose are used for osmotic balance [29] fructans and some other sugar alcohols, serve to maintain cell turgor during drought and salt stress, but these functions are limited to specific plant families [119]. Although various studies were conducted to explore the underlying roles of fructan in stress-tolerance strategies, many aspects still remain unclear. Therefore, it is crucial to develop experiments to obtain new insights into fructans’ roles under stress conditions to develop stress-alleviating strategies [31].

Another challenge is to compare the measurement protocols for NSCs among different labs working with different equipment and methodologies. However, Quentin et al., 2015 reported on the intra-lab reproducibility and precision of NSCs values, enabling the comparison of NSCs dynamics for different treatments within the same laboratory [114]. It is crucial for the researchers to develop standardized and reliable methods for sampling and NSCs measurement protocols to use across different laboratories and for diverse plant species as proposed by Landhäusser et al. (2018) [120]. Unfortunately, many laboratories still provide NSCs evaluations using other rough methods based on hydrochloric acid (HCl) digestion and spectrophotometric approaches and not based on the methods involved glucose conversion in NADH (see [121]).

Different studies provide evidence for the roles of NSCs in plant survival, growth, and recovery after stress conditions; however, other resources such as phosphorus and nitrogen may also show variations with the NSCs levels and may play equally important roles in plant recovery [122]. Therefore, for future research, it is a major challenge to design experiments that could investigate the specific functional roles of major stored resources and their interactions. Additionally, in plants, belowground symbionts such as mycorrhizal fungi are also significant consumers of NSCs [123], but the understanding regarding the allocation of NSCs to the belowground symbionts is minimal as explained by Schiestl-Aalto et al., 2019 [124]. Therefore, to demonstrate the carbon balance of a whole tree, it is important to design the experiment with belowground symbionts as a central focus.

Several studies have investigated the individual effects of abiotic stress in woody plants; however, in natural ecosystems, most stresses often co-occur, and these combined stresses are expected to become more frequent in the future [125]. A complete overview of the effects of all these combined stresses on the regulation of NSCs is still lacking. Considering the discussion summarized in this review, we recognized the urgent need to design the research experiment in the field and in controlled conditions to analyze the combined effects of abiotic stresses on the interplaying role of NSCs under varying stress conditions. Recently, due to the boost given by climate changes, some articles dealing with the combined effect of temperature and water deprivation on NSCs allocation have been published [126,127,128]. This will be helpful in evaluating the generalized roles of NSCs in ecological strategies and physiological processes under diverse climatic conditions [29]. Recent studies that involved the use of isotope tracking methods for NSCs experiments drew significant attention to exploring the organ-specific NSCs allocation within trees [129,130]. To solve the complicated dynamic nature of NSCs, future studies should prioritize the time series analysis with a collection of multi-year data on the abiotic stress onset, response, and recovery phase [131]. Other advanced metabolomics techniques along with standardized protocols could be used to accurately quantify the different types of NSCs in different tissues under stress conditions [132]. Another crucial challenge is to transfer the experimental findings into agriculture field practices by using strategies like targeted breeding to improve NSCs reserve and allocation, alongside the improved agronomic practices, which have the potential to enhance the stress tolerance of woody plant crops.

## 4. Materials and Methods

To explore the roles of NSCs under abiotic stress, the literature was filtered through a targeted search of peer-reviewed journals using databases such as Scopus, Web of Science, and Google Scholar to retrieve reviews, peer-reviewed articles, and book chapters for the selection of data published from 2000 to 2024. A list of keywords like non-structural carbohydrates, NSCs, and abiotic stress were applied to the following category journals: agronomy and crop science, forestry, horticulture, plant science, and miscellaneous to search for each dataset. Publications were selected based on their relevance to the topic and the publication date (2006–2024). Additionally, a manual search was performed for the articles cited in the selected paper, applying the same eligibility criteria as described before. Different eligibility criteria were applied for the inclusion and exclusion of the published material. The language of the document was restricted to English. Studies were considered for inclusion if they particularly address the roles of NSCs in woody plants and their roles under abiotic stresses including drought, salt, water logging, heat, and cold, presenting the original research and reviews in relevance to the review scope. This study excluded conference abstracts and studies that focused on non-plant systems or on the role of NSCs in biotic stresses without discussing their interaction with the abiotic stresses. Further, the title and abstracts were screened for relevance to the topic, resulting in 132 articles selected for the full-text review and included in the final analysis.

## 5. Conclusions

This study reports the recent knowledge on NSCs in plants under abiotic stress conditions trying to depict critical aspects of stress resilience in woody plants. A comprehensive understanding of the regulation of NSCs allocation, storage, and remobilization in plants is crucial to elucidate that how plants respond to abiotic stress conditions. However, the roles of NSCs under stress conditions involve a complex dynamic process influenced by various factors including species type, stress type, plant developmental stage, tissue type, time of sampling, plant symbiotic relations, and many others. Therefore, there are substantial gaps in understanding the NSCs behaviors across diverse biomes, species, and environmental stresses. Despite the significant advancement in the understanding of NSCs’ roles, further research with standardized methods is necessary, regarding the temporal regulation of NSCs metabolism and allocation under varying stress conditions and to fully elucidate the interactive mechanisms of NSCs signaling pathways with other metabolic pathways including secondary metabolites and hormonal signaling across diverse species in different ecological contexts. Moreover, there is also a gap in the knowledge of the impact of seasonal timing (summer vs. spring), intensity, and stress duration on the NSCs reserves. Therefore, research experiments using metabolomics techniques, isotope tracking, or targeted breeding with multiple stress responses are essential to develop new, effective strategies for climate change. Expanding the knowledge of NSCs allocation and mobilization in response to the growing impact of climate change will be key to developing strategies to enhance the resilience of woody plants to abiotic stresses and ensure the sustainability of the ecosystem with the growing impact of climate change.

## Data Availability

The original contributions presented in the study are included in the article, further inquiries can be directed to the corresponding author.
